# Functional Characterization of 11 Tentative Microneme Proteins in Type I RH Strain of *Toxoplasma gondii* Using the CRISPR-Cas9 System

**DOI:** 10.3390/ani14172543

**Published:** 2024-09-01

**Authors:** Zhi-Ya Ma, Xiao-Jing Wu, Chuan Li, Jin Gao, Yong-Jie Kou, Meng Wang, Xing-Quan Zhu, Xiao-Nan Zheng

**Affiliations:** 1 Laboratory of Parasitic Diseases, College of Veterinary Medicine, Shanxi Agricultural University, Jinzhong 030801, China; mzy16635048046@126.com (Z.-Y.M.); wuxiaojing2017@163.com (X.-J.W.); 15796845248@163.com (C.L.); jingao2022@163.com (J.G.); kouyongjie1997@163.com (Y.-J.K.); 2 State Key Laboratory for Animal Disease Control and Prevention, Key Laboratory of Veterinary Parasitology of Gansu Province, Lanzhou Veterinary Research Institute, Chinese Academy of Agricultural Sciences, Lanzhou 730046, China; wangmeng02@caas.cn

**Keywords:** *Toxoplasma gondii*, microneme protein, genes of interest (GOIs), CRISPR-Cas9, toxoplasmosis

## Abstract

**Simple Summary:**

*Toxoplasma gondii* is a zoonotic pathogenic apicomplexan parasite that infects approximately one third of the global population. In immunosuppressed individuals, the infection may be fatal. Current anti-toxoplasmosis drugs have low efficacy and cause side effects, and no vaccine is available to prevent human toxoplasmosis. To search for potential virulence genes of *T. gondii* with a view to serving as new drug or vaccine targets for toxoplasmosis, we selected 11 genes of interest (GOIs), which were tentative microneme proteins (MICs) with unknown functions, and applied the CRISPR-Cas9 system to construct 11 epitope tagging strains and gene knockout strains to explore their intracellular localization and biological functions. The results showed that nine tentative MICs were localized or partially localized in the microneme, one GOI was localized in endoplasmic reticulum, and another GOI was localized in the dense granule. Deletion of the 11 genes showed no significant effect on the in vitro growth and virulence of the tachyzoites of *T. gondii* type I RH strain. This study investigated the role of the 11 tentative *mic*s in *T. gondii* tachyzoites, which expanded the knowledge of the functionally unknown genes of *T. gondii*. In the future, roles of these genes in other life stages and other genotypes of *T. gondii* will be explored.

**Abstract:**

*Toxoplasma gondii*, a pathogenic apicomplexan parasite, infects approximately one third of the world’s population and poses a serious threat to global public health. Microneme proteins (MICs) secreted by the microneme, an apical secretory organelle of *T. gondii*, play important roles in the invasion, motility, and intracellular survival of *T. gondii*. In this study, we selected 11 genes of interest (GOIs) of *T. gondii*, tentative MICs predicted to be localized in micronemes, and we used the CRISPR-Cas9 system to construct epitope tagging strains and gene knockout strains to explore the localization and function of these 11 tentative MICs. Immunofluorescence assay showed that nine tentative MICs (TGME49_243930, TGME49_200270, TGME49_273320, TGME49_287040, TGME49_261710, TGME49_205680, TGME49_304490, TGME49_245485, and TGME49_224620) were localized or partially localized in the microneme, consistent with the prediction. However, TGME49_272380 and TGME49_243790 showed different localizations from the prediction, being localized in the endoplasmic reticulum and the dense granule, respectively. Further functional characterization of the 11 RHΔ*GOI* strains revealed that deletion of these 11 GOIs had no significant effect on plaque formation, intracellular replication, egress, invasion ability, and virulence of *T. gondii*. Although these 11 GOIs are not essential genes for the growth and virulence of tachyzoites of type I RH strain, they may have potential roles in other developmental stages or other genotypes of *T. gondii*. Thus, further research should be performed to explore the possible role of the nine *mic*s and the other two GOIs in other life cycle stages and other genotypes of *T. gondii*.

## 1. Introduction

*Toxoplasma gondii* is an opportunistic pathogenic apicomplexan parasite, which can infect many warm-blooded animals, including humans and birds [1,2]. In immunocompetent individuals, *T. gondii* forms latent bradyzoites-containing cysts, usually resulting in asymptomatic toxoplasmosis in the host. When hosts become immunosuppressed, these latent parasites are activated and transformed into tachyzoites, and invade other host cells, causing toxoplasmic encephalitis in immunosuppressed patients [3]. In addition, research suggests that a wide range of psychiatric disorders may be associated with *T. gondii* chronic latent infection, resulting in a public health burden [4]. Current anti-toxoplasmosis medications not only have low efficacy and adverse effects, but are also ineffective against chronic infection [5,6]. Therefore, there is an urgent need to identify new medication targets for toxoplasmosis.

Invasion of *T. gondii* into host cells is completed through multiple steps, including contact, attachment, secretion of associated proteins, and formation of a moving junction (MJ) [7,8]. Microneme proteins (MICs), secreted in a calcium-dependent manner from the *T. gondii* apical end, play important roles in the attachment of *T. gondii* to the host cells. Before invasion to host cells, *T. gondii* displays a low level of MICs expression [9,10]. When *T. gondii* begins to contact with host cells, the increased Ca^2+^ concentration stimulates the secretion of a large number of MICs stored in the apical part of *T. gondii* [9,10]. The high level of secreted MICs with transmembrane structural domains can be linked to aldolase of the *T. gondii* actin system, which is a key power source of motility and adhesion in *T. gondii*, resulting in the successful invasion of the host [11].

In *T. gondii*, MICs commonly function as complexes. TgMIC1/4/6 complex, the first identified complex in *T. gondii*, is involved in the invasion, pathogenesis, and immune evasion of *T. gondii* [12,13]. TgMIC1 contributes to the interaction with TgMIC4 and TgMIC6 and the complex targeting to the microneme [14]. TgMIC1 contributes to the secretion of TgMIC4 and TgMIC6. Knockout TgMIC1 and TgMIC6 disrupt the function of the entire complex and significantly reduced the invasion efficiency [15]. TgMIC2 and TgM2AP are also a complex, in which TgMIC2 is responsible for anchoring the host cell membrane and TgM2AP is responsible for the proper secretion and transport of TgMIC2 [16]. TgMIC2 expression was significantly decreased in the TgM2AP knockout strain, with an impaired invasion rate of more than 80% during invasion of host cells [17]. TgMIC3 and TgMIC8 can form an adhesion complex, which adheres to glycoproteins on the surface of host cells during the invasion of *T. gondii* [18]. Studies have shown that knockout of both TgMIC3 and TgMIC8 individually does not affect the other’s targeting to the microneme [15,19].

Given the important role of MICs in *T. gondii*, it is necessary to characterize tentative MICs and explore their role in the pathogenesis of *T. gondii*. Recently, many tentative MICs of *T. gondii* have been identified via hyperplexed localization of organelle proteins by isotopic tagging (hyperLOPIT) combined with proteomics [20]. However, the functions of most tentative MICs are still unknown. To understand the roles of tentative MICs in the pathogenesis of *T. gondii*, we chose 11 tentative *mic* genes with lower phenotype values, which suggest their involvement in *T. gondii* fitness. We evaluated their function in the pathogenicity of *T. gondii* type I RH strain by constructing knockout strains using the CRISPR-Cas9 system and a series of phenotypic experiments. Our data showed that deletion of these 11 genes of interest (GOIs) does not affect the normal growth in vitro and the virulence of *T. gondii* type I RH strain in vivo, suggesting that these 11 GOIs are not essential for the tachyzoite stage of *T. gondii* RH strain.

## 2. Materials and Methods

### 2.1. Bioinformatic Analysis of 11 Tentative MICs

In this study, the 11 GOIs were predicted to be tentative *mic* genes. Bioinformatic information of these GOIs were obtained from the *T. gondii* genome database (http://toxodb.org, accessed on 18 July 2023). The obtained genomic data included the number of exons, phenotypic value, molecular weight, transmembrane domains (TMHMM), and predicted signal peptide.

### 2.2. Mice

Female Kunming mice aged 6–8 weeks were purchased from the Experimental Animal Center of Lanzhou Veterinary Research Institute, Chinese Academy of Agricultural Sciences (Lanzhou, China). All mice were kept in specific-pathogen-free (SPF) conditions and had free access to commercial sterilized food and water. All mice were given one week to acclimatize to the environment before the experiments. Animal experiments were reviewed and approved by the Animal Ethics Committee of Lanzhou Veterinary Research Institute, Chinese Academy of Agricultural Sciences (Permit No. 2023-007).

### 2.3. Parasite Strains

*Toxoplasma gondii* tachyzoites used in this experiment included type I RHΔ*ku80* (referred as to RH) and constructed RHΔ*ku80*Δ*GOI* (referred as to RHΔ*GOI*) knockout strains, which were grown in monolayers of human foreskin fibroblasts (HFFs, ATCC, Manassas, VA, USA) cell. The HFFs cell were cultured in Dulbecco’s Modified Eagle Medium (DMEM) supplemented with 10% fetal bovine serum (FBS), 10 mM HEPES (pH 7.2), 100 µg/mL streptomycin, and 100 U/mL penicillin at 37 °C with 5% CO_2_ [21,22]. When cells were heavily infected with parasites, samples were scraped off using a cell scraper and a 27-gauge needle to release all tachyzoites and were then filtered through a 5-μm polycarbonate membrane.

### 2.4. Construction of Epitope Tagging Strains

To obtain the subcellular localization of tentative MICs at the tachyzoite stage, the 11 tentative MICs were C-terminally tagged with hemagglutinin (HA) epitope. Specifically, the CRISPR-Cas9 plasmids targeting the C-terminus of the GOI were obtained using specific primers as described previously [23,24]. The homologous fragment with 42 bp homologous arms, including a 6 × HA fragment and a dihydrofolate reductase (DHFR) fragment, was amplified from the pLIC-6HA-DHFR plasmid. The CRISPR-Cas9 plasmids and the homologous fragment of each GOI were co-transfected into RHΔ*ku80* tachyzoites. After selection with pyrimethamine, the positive single clones were verified by Polymerase Chain Reaction (PCR). All primers used are shown in Supplementary Table S1.

### 2.5. Construction of Gene Knockout Strains

CRISPR-Cas9-mediated homologous recombination was used to obtain GOI knockout strains as previously described [21]. A specific CRISPR-Cas9 plasmid was obtained by using the small guide RNA (SgRNA) of GOI to replace the SgRNA of uracil phosphoribosyl transferase (UPRT) in pSAG1:CAS9-U6-SgUPRT plasmid. To construct a 5′UTR-DHFR-3′UTR homologous plasmid of a GOI, the homologous fragments, the DHFR fragment, and the PUC19 fragment were amplified from *T. gondii* genomic DNA, the pUPRT-DHFR-D plasmid, and the PUC19 plasmid, respectively. These fragments were fused using the Clone Express II one-step cloning kit (Vazyme, Nanjing, China). The 5′UTR-DHFR-3′UTR fragments amplified from the valid plasmid along with the positive CRISPR-Cas9 plasmids were co-transfected into freshly tachyzoites as previously described [21]. The single clones were drug selected with 3 μM pyrimethamine, screened in a 96-well plate with limited dilution, and identified by PCR. All primers used are shown in Supplementary Table S2.

### 2.6. Immunofluorescence Assay and Western Blotting Analysis

Immunofluorescence assay (IFA) was used to determine the localization of 11 tentative MICs in *T. gondii* type I RH strain as previously described [25]. After freshly egressed tachyzoites of epitope tagging strains were added to HFF monolayers for 24 h, samples were fixed with 4% paraformaldehyde (PFA) for 30 min, permeabilized with 0.1% Triton X-100 for 30 min, and blocked with 3% BSA. Subsequently, the samples were co-incubated with primary antibody mouse anti-HA (1:500) (Thermo Fisher Scientific, Waltham, MA, USA), rabbit anti-IMC1 (1:500), rabbit anti-MIC2 (1:500), or rabbit anti-GRA12 for 2 h at 37 °C, and then incubated with the secondary antibodies, Alexa Fluor 488 goat anti-rabbit IgG (H + L) (1:1000) and Alexa Fluor 594 goat anti-mouse IgG (H + L) (1:1000) (Thermo Fisher Scientific, Waltham, MA, USA), for 1 h at 37 °C. Nuclei were stained with 4’, 6-diamidino-2-phenylindole (DAPI) after incubation with secondary antibodies. Each procedure was followed by four washes with PBS. Images were acquired using a Leica confocal microscope system (TCS SP8, Leica, Munich, Germany) after the final wash.

For Western Blotting analysis, total protein was extracted by incubation with RIPA buffer on ice for 1 h after twice washes with PBS. The protein samples were separated by SDS-PAGE and transferred onto a polyvinylidene fluoride (PVDF) membrane. Antibodies used in Western Blotting included rabbit anti-aldolase (1:500), rabbit anti-HA (1:500), and goat anti-rabbit HRP (1:5000). The signals were detected using an ECL chemiluminescence kit.

### 2.7. Parasite Plaque Assay

To examine the ability of 11 RHΔ*GOI* strains to form plaque, 300 freshly egressed RHΔ*GOI* and RH tachyzoites were given access to HFF monolayers grown in 12-well cell plates. After being cultured for 7 days at 37 °C with 5% CO_2_, the culture medium was removed, and samples were fixed with 4% PFA for 30 min and stained with 0.2% crystal violet for 30 min at room temperature. The number and size of plaques were analyzed with ImageJ 1.8 software.

### 2.8. Invasion Assay

IFA was used to explore the effect of GOIs deletion on the invasion efficiency. To begin, 1 × 10^6^ freshly egressed RHΔ*GOI* and RH tachyzoites were used to invade HFF monolayers at 37 °C with 5% CO_2_. After invasion for 30 min, 4% PFA was added for fixation for 30 min. Then, samples were respectively incubated with primary mouse anti-SAG1 antibody (1:500) and secondary Alexa Fluor 594 goat anti-mouse IgG (H + L) (1:1000). After washing with PBS three times, the samples were permeabilized with 0.1% Triton X-100 and sequentially incubated with primary antibody rabbit anti-IMC1 (1:500) and secondary antibody Alexa Fluor 488 goat anti-rabbit IgG (H + L) (1:1000). Non-invaded tachyzoites were colored red and total tachyzoites were colored green.

### 2.9. Intracellular Replication and Egress Assay

To explore the role of the 11 tentative MICs in the intracellular replication and egress of *T. gondii*, we performed tachyzoite intracellular replication and egress experiments as described previously [23]. To begin, 1 × 10^5^ freshly egressed RHΔ*GOI* and RH tachyzoites were added into HFF monolayers of 12-well cell plates for invasion. The extracellular tachyzoites were removed after 1 h, and the incubation was continued for 23 h. Samples were fixed with 4% PFA for 30 min after removing the culture medium. Primary mouse anti-SAG1 antibody (1:500) and secondary Alexa Fluor 488 goat anti-mouse IgG (H + L) (1:1000) were used to incubate the samples sequentially. The numbers of tachyzoites within at least 100 parasitophorous vacuoles (PVs) were counted under a fluorescence microscope.

In egress assays for RHΔ*GOI* and RH strains, samples were continuedly incubated for 36 h after removing the noninvaded tachyzoites. The samples were stimulated with 3 μM calcium ionophore A23187 (Sigma, Burlington, MA, USA) for 2.5 min and fixed with 4% PFA. The egress efficiency was determined by calculating the egressed PVs-to-all PVs ratio under the microscope, as previously described [26].

### 2.10. Virulence Assessment in Mice

To explore the effect of deletion of the 11 tentative *mic* genes on the virulence of *T. gondii* type I RH strain, 6- to 8-week-old Kunming female mice were intraperitoneally (i.p.) injected with 100 freshly egressed RHΔ*GOI* and RH tachyzoites. The mice were monitored twice daily, and their survival was recorded. Once the humane endpoint was reached, they were euthanized immediately to reduce unnecessary suffering [27].

### 2.11. Statistical Analysis

Statistical comparisons of the obtained data were performed using GraphPad Prism version 8.0.l. Each experiment was repeated three times independently. Two-tailed, unpaired Student *t*-test and one-way ANOVA were used to perform analyses of variance between two, three, or more groups. Results are shown as mean ± standard deviation (SD). When *p* < 0.05, the difference was considered statistically significant between the two groups of data.

## 3. Results

### 3.1. Bioinformatic Characteristics of 11 Tentative mic Genes in T. gondii

In this study, 11 GOIs tentatively predicted as microneme proteins were investigated. The bioinformatic characteristics of the 11 tentative *mic* genes are shown in Table 1. The phenotype values of 11 tentative *mic* genes range from −1.1 (TGME49_287040) to 1.77 (TGME49_205680). These GOIs contain different numbers of exons (1~8) and code different sizes (23.448~101.348 kDa) of proteins. Except for TGME49_243930, TGME49_200270, TGME49_287040, and TGME49_261710, the seven tentative MICs possess transmembrane domains. Six tentative MICs possess signal peptides, except TGME49_243790, TGME49_261710, TGME49_272380, TGME49_304490, and TGME49_224620.

### 3.2. Subcellular Localization of 11 Tentative MICs

**Figure 1:**
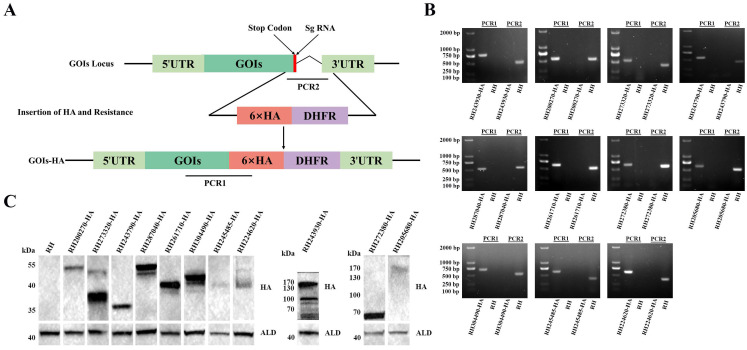
Construction of epitope tagging strains. (**A**) Schematic diagram of constructing epitope-tagging strains by C-terminal tagging using the CRISPR-Cas9 system. UTR, untranslated region. GOIs, genes of interest. HA, hemagglutinin. DHFR, dihydrofolate reductase. (**B**) Successful construction of epitope tagging strains was verified by PCRs. PCR1 and PCR2 were used to verify the correct insertion of the 6 × HA and DHFR fragment. (**C**) Western Blotting further confirmed the expression of the 6 × HA-tagged proteins. Aldolase (ALD) was used as a loading control.

To explore the subcellular localization of the 11 tentative MICs in *T. gondii* type I RH strain, we inserted a 6 × HA tag at the C-terminus of MICs using CRISPR-Cas9 (Figure 1A). The HA tagged strains were demonstrated by PCR and sequencing. PCR1 showed that a fragment of ~500–750 bp was amplified in epitope tagging strains, demonstrating successful insertion of the 6 × HA, whereas no fragment was amplified in RH strain. PCR2 showed that the 6 × HA and DHFR sequence was inserted into the locus near the STOP codon of GOIs in epitope tagging strains, in which the C-terminus sequence of GOIs could not be amplified within 45 s; the corresponding fragments could be amplified in the RH strain (Figure 1B). Western Blotting results further verified the successful expression of the tagged proteins. The specific bands of RH200270-HA, RH273320-HA, RH287040-HA, and RH304490-HA were consistent with those predicted in ToxoDB. The bands of RH261710-HA, RH243930-HA, and RH205680-HA were larger than predicted, while RH243790-HA, RH245485-HA, RH224620-HA, and RH272380-HA had slightly smaller bands than those predicted. Moreover, there were other bands except the predicted bands in RH273320-HA, RH287040-HA, RH304490-HA, and RH243930-HA (Figure 1C).

**Figure 2:**
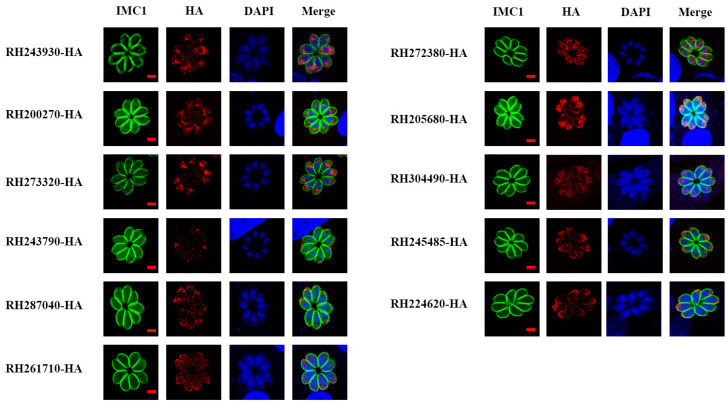
Subcellular localization of 11 tentative MICs in the tachyzoite stage of *T. gondii* type I RH strain. Epitope-tagging strains were used to infect HFF monolayers for 24 h. Samples were stained with two antibodies, including anti-IMC1 (green) and anti-HA (red), and nuclei were stained with DAPI (blue). Scale bars, 2 μm. IMC1, inner membrane complex 1. HA, hemagglutinin. DAPI, 4’, 6-diamidino-2-phenylindole.

The 11 GOIs were predicted to be localized in micronemes via hyperplexed localization of organelle proteins by isotopic tagging (hyperLOPIT) combined with proteomics [20]. In this study, IFA results showed that RH273320-HA, RH205680-HA, RH245485-HA, and RH224620-HA were localized in micronemes, and RH243930-HA, RH200270-HA, RH287040-HA, RH261710-HA, and RH304490-HA were partially localized in micronemes, consistent with the prediction by hyperLOPIT. Co-localization with the microneme marker MIC2 confirmed that these nine proteins were MICs (Figure S1). RH272380-HA was localized in the endoplasmic reticulum and RH243790-HA was localized to the dense granule (Figure 2 and Figure S1).

### 3.3. Successful Construction of 11 GOIs Knockout Strains

**Figure 3:**
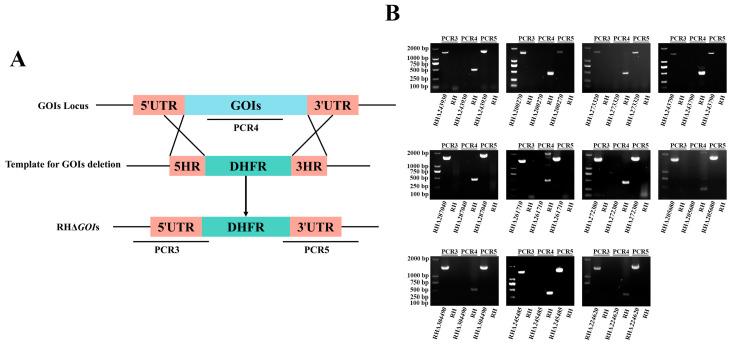
Construction of RHΔ*GOI* strains. (**A**) Schematic diagram of the construction of GOI deletion strains by CRISPR-Cas9-mediated homologous recombination. UTR, untranslated region. GOIs, genes of interest. HR, homologous arm. DHFR, dihydrofolate reductase. (**B**) Successful construction of RHΔ*GOI* strains was verified by PCRs. PCR3 and PCR5 were used to verify the correct insertion of the 5′UTR-DHFR-3′UTR homologous fragment, and PCR4 was used to confirm the successful deletion of GOI.

To study the biological functions of these 11 GOIs in *T. gondii* type I RH strain, coding regions of GOIs were replaced with the corresponding 5′UTR-DHFR-3′UTR homologous fragment to disrupt GOIs using CRISPR-Cas9 system (Figure 3A). The single clones, obtained by limited dilution and pyrimethamine screening, were verified by PCRs. PCR4 showed that a fragment (~500 bp) in the coding region of the GOIs was amplified in the RH strain, while no fragment was amplified in the knockout strains (Figure 3B). PCR3 and PCR5 of the knockout strains showed the amplified ~1000–1500 bp fragments, but not in the RH strain. The PCR result demonstrated the successful insertion of the 5′UTR-DHFR-3′UTR homologous fragment into the target genes (Figure 3B). Taken together, these results demonstrate that we successfully constructed 11 knockout strains using the CRISPR-Cas9 system.

### 3.4. Deletion of 11 GOIs Does Not Affect T. gondii Growth

**Figure 4:**
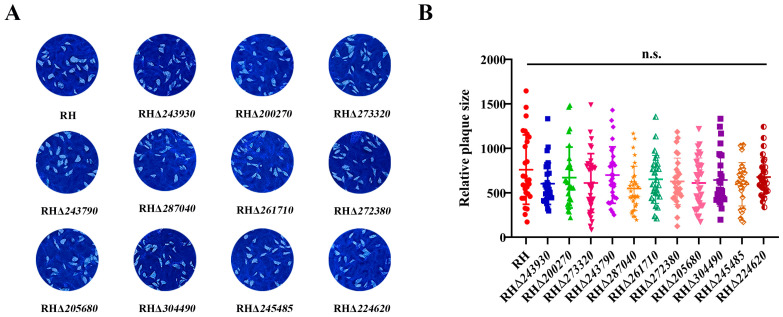
Plaque assay of RH and 11 RHΔ*GOI* strains. (**A**) Representative plaques formed by the 11 RHΔ*GOI* strains and the RH strain. (**B**) There was no significant difference in the relative size of plaques formed by the RHΔ*GOI* strains and RH strain (*p* > 0.05). n.s., not significant.

To further investigate the effect of deleting the 11 GOIs on the overall growth of *T. gondii*, we performed a plaque assay. After 7 days of growth in HFF monolayers, the average plaque area formed by the 11 RHΔ*GOI* strains (RHΔ*243930*, RHΔ*200270*, RHΔ*273320*, RHΔ*243790*, RHΔ*287040*, RHΔ*261710*, RHΔ*272380*, RHΔ*205680*, RHΔ*304490*, RHΔ*245485*, and RHΔ*224620*) were not significantly different from that of the RH strain (*p* > 0.05) (Figure 4). These results suggest that the deletion of the 11 GOIs may not affect the in vitro lytic cycle of *T. gondii*.

**Figure 5:**
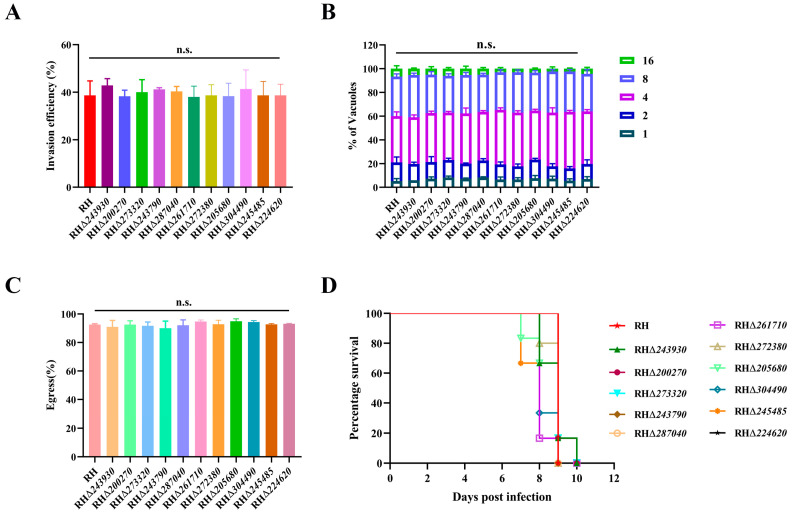
Invasion efficiency, intracellular replication, and egress efficiency assays of 11 RHΔ*GOI* strains in vitro*,* and virulence assays of 11 RHΔ*GOI* strains in vivo. (**A**) There was no significant difference between the 11 RHΔ*GOI* strains and the RH strain in terms of invasion efficiency (*p* > 0.05). (**B**) No significant difference in intracellular replicative ability between the 11 RHΔ*GOI* strains and the RH strain (*p* > 0.05). Freshly egressed tachyzoites of RHΔ*GOI* strains and the RH strain were used to infect HFF monolayers for 24 h, and at least a total of 100 PVs containing 1, 2, 4, 8, or 16 tachyzoites were counted. (**C**) The egress efficiency of the 11 RHΔ*GOI* strains and the RH strain were determined 36 h after infection in HFF monolayers, and the results showed no significant difference (*p* > 0.05). (**D**) Survival curve of Kunming mice showed no significant difference in RH infected group and 11 RHΔ*GOI* infected group. Kunming mice (6 mice per group) were injected intraperitoneally (i.p.) with 100 tachyzoites of different strains. n.s., not significant.

Next, we determined the invasion, intracellular replication, and egress abilities of 11 RHΔ*GOI* strains in vitro. To explore the invasion ability of the 11 RHΔ*GOI* strains, freshly egressed RHΔ*GOI* and RH tachyzoites were allowed to invade HFF monolayers for 30 min, and their invasion efficiency was calculated by IFA. The results showed that there was no significant difference between the 11 RHΔ*GOI* strains and the RH strain in terms of invasion efficiency (*p* > 0.05), indicating that deletion of these 11 GOIs did not affect the ability of *T. gondii* to invade cells in vitro (Figure 5A). Tachyzoites of the 11 RHΔ*GOI* strains and RH strain were grown in HFF monolayers for 24 h, and the numbers of tachyzoites within PVs were counted. The results showed that there was no significant difference between the 11 RHΔ*GOI* strains and RH strain in terms of intracellular replication (*p* > 0.05) (Figure 5B). Tachyzoites in PVs were stimulated with calcium ionophore after 36 h infection, and the egress efficiency was observed using the microscope. The results showed that most tachyzoites egressed within 2.5 min, with no significant difference in egress efficiency between the 11 RHΔ*GOI* strains and RH strain (*p* > 0.05) (Figure 5C). These results suggest that the deletion of 11 GOIs does not affect the normal growth and lytic cycle of *T. gondii* in vitro.

### 3.5. Deletion of 11 GOIs Does Not Affect the Virulence of T. gondii

To explore whether these 11 GOIs play a role in the virulence of *T. gondii* type I RH strain, the Kunming mice (six mice per group) were intraperitoneally (i.p.) injected with 100 freshly egressed tachyzoites of RHΔ*GOI* strains or RH strain. The results showed that all mice reached the human endpoint within 7–10 days. Thus, there was no significant difference in virulence between RHΔ*GOI* strains and RH strain (Figure 5D).

## 4. Discussion

*T. gondii* is a highly successful opportunistic pathogenic parasite. Although *T. gondii* infection may not show obvious clinical symptoms in immunocompetent populations, toxoplasmosis can be fatal in immunosuppressed populations. Current anti-toxoplasmosis drugs, such as pyrimethamine and sulfadiazine, have many side effects and no significant effect against the *T. gondii* bradyzoite [28,29,30]. In addition, there are no drugs available to treat congenital toxoplasmosis [31]. Therefore, it is very important to develop a vaccine with strong immune protection against toxoplasmosis. In recent years, many *T. gondii* antigens, such as MICs, rhoptry proteins (ROPs), dense granule proteins (GRAs), and excretory-secretory antigens (ESAs) have been studied in attempts to develop vaccines against *T. gondii* [32,33]. However, so far, no effective vaccine against human toxoplasmosis has been developed. To expand new target proteins or genes to develop anti-toxoplasmosis vaccines, in the present study, 11 tentative *mic* genes, predicted to be localized in micronemes and with unknown function, were selected and explored whether their deletion would affect the growth and virulence of *T. gondii*.

Gene knockout strains and epitope-tagging strains were successfully constructed using the CRISPR-Cas9 system. IFA showed that among the 11 tentative *mic* genes, nine genes (RH243930-HA, RH200270-HA, RH273320-HA, RH287040-HA, RH261710-HA, RH205680-HA, RH304490-HA, RH245485-HA, and RH224620-HA) were localized or partially localized in the microneme, the same as the HyperLOPIT prediction [20]. However, two tentative MICs showed different localization from the prediction in ToxoDB. RH272380-HA was localized in the endoplasmic reticulum and RH243790-HA was localized in the dense granule. Further research is needed to determine which type of protein they belong to.

Western Blotting of epitope-tagging RH strains showed detectable expression of the 11 GOIs. The results showed the expected band sizes of four *mic* proteins; however, the remaining five *mic* proteins and RH272380-HA and RH243790-HA had bands that were either larger or slightly smaller than predicted. In addition, there were other bands except the predicted bands in RH273320-HA, RH287040-HA, RH304490-HA, and RH243930-HA. These size discrepancies or extra bands observed in Western Blotting may be due to degradation, posttranslational modifications of these proteins, or the inaccurate predictions given in ToxoDB.

Micronemes, one of the unique organelles of the apicomplexan parasite, play enormous roles in parasite invasion and motility [34]. TgAMA1, which is an apical membrane antigen secreted by *T. gondii*, forms MJ when interacting with TgRON2, TgRON4, TgRON5, and TgRON8 secreted by *T. gondii* during invasion. This interaction is the sign and basis of active invasion [35,36,37]. The claudin-like apicomplexan microneme protein (CLAMP) is an important invasion factor of *T. gondii* and is required for the asexual replication cycle of malaria parasite *Plasmodium falciparum* [38]. Conditional knockdown of CLAMP significantly reduced *T. gondii* invasion efficiency and completely blocked the *P. falciparum* replication in the asexual cycle [38]. CLAMP-Linked Invasion Protein (CLIP) is a newly identified MICs. Knockdown of CLIP in *T. gondii* prevents the formation of plaque and invasion of host cells, resulting in a stalled in vitro lytic cycle [7]. Although many MICs have been shown to be key invasion factors and virulence factors of *T. gondii*, deletion of these nine *mic* genes and other two GOIs in this study did not affect the invasion ability and virulence of *T. gondii* type I RH strain. No significant difference was observed between RH strain and gene knockout strains in terms of in vitro plaque formation ability and egress ability. These results showed that these 11 GOIs were not essential for the tachyzoite stage of RH strain.

Infection, pathogenesis, and transmission of *T. gondii* depend on different stages in hosts. The asexual tachyzoites and bradyzoites are respectively responsible for acute and chronic *T. gondii* infection. The oocysts shed in cat feces are also infective when they become sporulated. Although these nine *mic* genes and the two GOIs showed no significant role in growth and virulence of *T. gondii* in the tachyzoite stage, it cannot be ruled out that they may play a role in other developmental stages of *T. gondii*. Different genotypes of *T. gondii* show different virulence [39]. The RH strain is the representative type I strain of high virulence, causing rapid death of mice within two to three weeks after infection with one viable RH tachyzoite; however, type II and type III strains, which are represented by ME49 strain and VEG strain, respectively, are moderately or low virulent for mice [40]. Many genes are strain/genotype-specific, such as GRA15 [41]. In this study, although the deletion of 11 GOIs had no significant impact on the growth and virulence of the RH tachyzoites, they may play a role in type II or type III strains of *T. gondii*. Further studies are warranted to explore the possible roles of these GOIs in other life cycle stages and/or other genotypes of *T. gondii*.

## 5. Conclusions

In this study, we successfully constructed epitope-tagging and gene deletion strains of 11 tentative MICs in *T. gondii* type I RH strain. Our data revealed the localization of the 11 GOIs in RH tachyzoites, confirming that nine tentative MICs were localized or partially localized in the microneme, one GOI was localized in the endoplasmic reticulum and the other GOI was localized in the dense granule. Disruption of the 11 GOIs had no significant impact on the plaque formation, intracellular replication, egress efficiency, invasion efficiency, and virulence in *T. gondii* type I RH tachyzoites. Although these nine *mic* genes and two GOIs are not essential genes for the tachyzoite stage of *T. gondii* type I RH strain, it is necessary to continue exploring their possible roles in other life cycle stages and other genotypes of *T. gondii*.

## Figures and Tables

**Table 1:** Bioinformatic features of the tentative microneme proteins (MICs) of *Toxoplasma gondii*.

Gene ID	Product Description	Exons	Phenotype Value	Mol wt (kDa)	TMHMM *	Predicted Signal Peptide
TGME49_243930	hypothetical protein	6	0.25	101.348	no	yes
TGME49_200270	PAN/Apple domain-containing protein	1	0.93	39.104	no	yes
TGME49_273320	hypothetical protein	6	0.27	25.589	yes	yes
TGME49_243790	SAG-related sequence SRS33	1	−0.5	31.839	yes	no
TGME49_287040	hypothetical protein	3	−1.1	36.230	no	yes
TGME49_261710	ankyrin repeat-containing protein	8	1.7	23.448	no	no
TGME49_272380	hypothetical protein	3	1.71	61.026	yes	no
TGME49_205680	hypothetical protein	5	1.77	79.066	yes	yes
TGME49_304490	hypothetical protein	5	1.49	31.978	yes	no
TGME49_245485	microneme protein MIC9	1	1.59	31.558	yes	yes
TGME49_224620	hypothetical protein	8	1.02	32.578	yes	no

* Prediction of transmembrane helices was performed using the TMHMM program version 2.0.

## Data Availability

The original contributions presented in this study are included in the article. Further inquiries can be directed to the corresponding authors.

## Supplementary Materials

The following supporting information can be downloaded at: https://www.mdpi.com/article/10.3390/ani14172543/s1, Table S1: Primers used in the construction of the epitope-tagging *T. gondii* strains; Table S2: Primers used in the construction of *T. gondii* RH∆*GOI* strains; Figure S1: Co-localization figures.
