# Supplementary material for: Functional Characterization of 11 Tentative Microneme Proteins in Type I RH Strain of Toxoplasma gondii Using the CRISPR-Cas9 System
Source: Animals (Basel). 2024 Sep 1;14(17):2543. doi: 10.3390/ani14172543 (PMC11394663; doi:10.3390/ani14172543)
Supplement: Supplementary file 1 [file animals-14-02543-s001.zip › Supplementary Table 1.pdf]

Supplementary Table S1 Primers used in the construction of the epitope tagging *T. gondii* strains.

Primer	Sequence (5′-3′)	Use
sgRNA-TGME49_243930-Tag-Fw	CTGTGACGGCTTTGTAAAAGGTTTTAGAGCTAGAAATAGC	Construct the CRISPR plasmid for tagging TGME49_243930 with 6×HA
sgRNA-Rv	AACTTGACATCCCCATTTAC	Construct the CRISPR plasmid for tagging TGME49_243930 with 6×HA
TGME49_243930-HR-Fw	GGCACACTTGCCGTGGCTGGTTTGGTTGCTGTGACGGCTTTGGCTAGCAAGGGCTCGGG	Amplify 6HA-DHFR fragment with the homologous fragment of *TGME49_243930*
TGME49_243930-HR-Rv	TCATGAGCAGCGTAAACAGCAGTGCTTTTCGCGATGCCGCTTATACGACTCACTATAGG	Amplify 6HA-DHFR fragment with the homologous fragment of *TGME49_243930*
PCR1-Rv	ATTATACCCGTGTGTTACG	Detect the insert 6×HA fragment
PCR1/2-TGME49_243930-Fw	TGGTTAGAATGACCCGTGAG	Detect the replacement of C-terminal TGME49_243930 by 6×HA fragment
PCR2-TGME49_243930-Rv	TGAGCAGCGTAAACAGCA	Detect the replacement of C-terminal TGME49_243930 by 6×HA fragment
sgRNA-TGME49_200270-Tag-Fw	GCTCGTCATTTCTAAGAATTGTTTTAGAGCTAGAAATAGC	Construct the CRISPR plasmid for tagging TGME49_200270 with 6×HA
TGME49_200270-HR-Fw	GCTCAAACTGATGCGAACTCCAAAGCAGGCGATATCACATGCGCTAGCAAGGGCTCGGG	Amplify 6HA-DHFR fragment with the homologous fragment of *TGME49_200270*
TGME49_200270-HR-Rv	GAGGATCCGCTCAAATAACAATGATTCCGCTACGTTTCCGAAATACGACTCACTATAGG	Amplify 6HA-DHFR fragment with the homologous fragment of *TGME49_200270*
PCR1/2-TGME49_200270-Fw	CAAATACAACCGTCAAGGC	Detect the replacement of C-terminal TGME49_200270 by 6×HA fragment
PCR2TGME49_200270-Rv	AGTTCCACTCACCCAAGC	Detect the replacement of C-terminal TGME49_200270 by 6×HA fragment
sgRNA-TGME49_273320-Tag-Fw	GAGAGCGGCAGGCTGTGAAAGTTTTAGAGCTAGAAATAGC	Construct the CRISPR plasmid for tagging TGME49_273320 with 6×HA
TGME49_273320-HR-Fw	TCGGGCCTCCCGCCTCCGTCTCCGAAAGAGAGCGGCAGGCTGGCTAGCAAGGGCTCGGG	Amplify 6HA-DHFR fragment with the homologous fragment of *TGME49_273320*
TGME49_273320-HR-Rv	TCCGCTAGACTGTCACCCATCGCTTGACGCGCGCATCCTTTTATACGACTCACTATAGG	Amplify 6HA-DHFR fragment with the homologous fragment of *TGME49_273320*
PCR1/2-TGME49_273320-Fw	TTCGCAGGTGGATTCTCG	Detect the replacement of C-terminal TGME49_273320 by 6×HA fragment
PCR2-TGME49_273320-Rv	CTGGCTTTCCTTGCTTCATT	Detect the replacement of C-terminal TGME49_273320 by 6×HA fragment
sgRNA-TGME49_243790-Tag-Fw	AATGACAGGCTGAGGAAACGGTTTTAGAGCTAGAAATAGC	Construct the CRISPR plasmid for tagging TGME49_243790 with 6×HA
TGME49_243790-HR-Fw	CTGGCTCTGGCGGGGCTTCTTGCGACAGCCGGAATGACAGGCGCTAGCAAGGGCTCGGG	Amplify 6HA-DHFR fragment with the homologous fragment of *TGME49_243790*
TGME49_243790-HR-Rv	ACTACGCTTTTTTCTGCCAGCCTTCTCGCGGGCTTCCCTCGTATACGACTCACTATAGG	Amplify 6HA-DHFR fragment with the homologous fragment of *TGME49_243790*
PCR1/2-TGME49_24379-Fw	CACCGTACCCGTCGTTCA	Detect the replacement of C-terminal TGME49_243790 by 6×HA fragment
PCR2-TGME49_243790-Rv	CTCCCAGAAACAGCAAGTCC	Detect the replacement of C-terminal TGME49_243790 by 6×HA fragment
sgRNA-TGME49_287040-Tag-Fw	AATGCAGCGACACACTCCTGGTTTTAGAGCTAGAAATAGC	Construct the CRISPR plasmid for tagging TGME49_287040 with 6×HA
TGME49_287040-HR-Fw	CGTGCCTTCGTGACTGCTGACCTCAAGCAAAGAATGAGTGCTGCTAGCAAGGGCTCGGG	Amplify 6HA-DHFR fragment with the homologous fragment of *TGME49_287040*

TGME49_287040-HR-Rv	ACCTAGTTCGTCGCAATGGCAATACTAGGTCAGAGTCCGCAGATACGACTCACTATAGG	Amplify 6HA-DHFR fragment with the homologous fragment of *TGME49_287040*
PCR1/2-TGME49_287040-Fw	CTCTGACTGGCGGCTTTG	Detect the replacement of C-terminal TGME49_287040 by 6×HA fragment
PCR2-TGME49_287040-Rv	TCACGGTCTGGCAGGGTA	Detect the replacement of C-terminal TGME49_287040 by 6×HA fragment
sgRNA-TGME49_261710-Tag-Fw	ACATCAAACTCGCCACAAAGGTTTTAGAGCTAGAAATAGC	Construct the CRISPR plasmid for tagging TGME49_261710 with 6×HA
TGME49_261710-HR-Fw	GTCGACGCGGGTAAAGCGGAGCTGAACCCTGCTCAAGCTTCTGCTAGCAAGGGCTCGGG	Amplify 6HA-DHFR fragment with the homologous fragment of *TGME49_261710*
TGME49_261710-HR-Rv	ATTTACCGGTCGGCACGGCAGCCGGTGCAGACTCTGCCTCTTATACGACTCACTATAGG	Amplify 6HA-DHFR fragment with the homologous fragment of *TGME49_261710*
PCR1/2-TGME49_261710-Fw	TGGCAGCGATGGTGCGAAAT	Detect the replacement of C-terminal TGME49_261710 by 6×HA fragment
PCR2-TGME49_261710-Rv	CCACGAACCAACCAACGGTATTG	Detect the replacement of C-terminal TGME49_261710 by 6×HA fragment
sgRNA-TGME49_272380-Tag-Fw	CGGTTTTGTGACGGGTTGACGTTTTAGAGCTAGAAATAGC	Construct the CRISPR plasmid for tagging TGME49_272380 with 6×HA
TGME49_272380-HR-Fw	TGCTGGTTCACGGCAGCTGCACTTATCGGTTTTGTGACGGGTGCTAGCAAGGGCTCGGG	Amplify 6HA-DHFR fragment with the homologous fragment of *TGME49_272380*
TGME49_272380-HR-Rv	CCTGTGGCTGCCGGAAGGTGGGTCCCCCACGTAATCCCTGTCATACGACTCACTATAGG	Amplify 6HA-DHFR fragment with the homologous fragment of *TGME49_272380*
PCR1/2-TGME49_272380-Fw	GGCAACCGATGCTACCGAACA	Detect the replacement of C-terminal TGME49_272380 by 6×HA fragment
PCR2-TGME49_272380-Rv	CGCTGAGGCACCCTTAACACG	Detect the replacement of C-terminal TGME49_272380 by 6×HA fragment
sgRNA-TGME49_205680-Tag-Fw	CGACGTAACTTCCGCTACTAGTTTTAGAGCTAGAAATAGC	Construct the CRISPR plasmid for tagging TGME49_205680 with 6×HA
TGME49_205680-HR-Fw	GGCCGAAGTCGTCAGAATGACGCTCCGGATCAGGTCGAATCAGCTAGCAAGGGCTCGGG	Amplify 6HA-DHFR fragment with the homologous fragment of *TGME49_205680*
TGME49_205680-HR-Rv	CAGGGCAGTGAAGTTTGGTCTTTGCTGTTTCGAAGCCCTTAGATACGACTCACTATAGG	Amplify 6HA-DHFR fragment with the homologous fragment of *TGME49_205680*
PCR1/2-TGME49_205680-Fw	TTTGGTAACCTGTGAAATGTGGCG	Detect the replacement of C-terminal TGME49_205680 by 6×HA fragment
PCR2-TGME49_205680-Rv	AAGTGGAATGGCGTGAGACGGT	Detect the replacement of C-terminal TGME49_205680 by 6×HA fragment
sgRNA-TGME49_304490-Tag-Fw	CGTTGCAAGCCTCTCGTGACGTTTTAGAGCTAGAAATAGC	Construct the CRISPR plasmid for tagging TGME49_304490 with 6×HA
TGME49_304490-HR-Fw	GATCTATCGCTGTTCCAAAACGCGGACGTTGCAAGCCTCTCGGCTAGCAAGGGCTCGGG	Amplify 6HA-DHFR fragment with the homologous fragment of *TGME49_304490*
TGME49_304490-HR-Rv	TTGAAGCCTGTTCTAACTGTGGTCACCGGCTCCTTGCCGGTCATACGACTCACTATAGG	Amplify 6HA-DHFR fragment with the homologous fragment of *TGME49_304490*
PCR1/2-TGME49_304490-Fw	TCGTGTCTGTCCGCTTGAATCCT	Detect the replacement of C-terminal TGME49_304490 by 6×HA fragment
PCR2-TGME49_304490-Rv	CGCTGGCATCGCGTTATGAA	Detect the replacement of C-terminal TGME49_304490 by 6×HA fragment
sgRNA-TGME49_245485-Tag-Fw	GGAATGTATTGATTGCCGTGGTTTTAGAGCTAGAAATAGC	Construct the CRISPR plasmid for tagging TGME49_245485 with 6×HA
TGME49_245485-HR-Fw	TCTAACGACGCTCTGTATGCACACGATTTTGAAGGAATGTATGCTAGCAAGGGCTCGGG	Amplify 6HA-DHFR fragment with the homologous fragment of *TGME49_245485*
TGME49_245485-HR-Rv	CTATGTCGCAATAATCCCCAAATACTGAGCATGCCACCACACATACGACTCACTATAGG	Amplify 6HA-DHFR fragment with the homologous fragment of *TGME49_245485*
PCR1/2-TGME49_245485-Fw	TAACAACGGAGCAGGGAGAAAGGT	Detect the replacement of C-terminal TGME49_245485 by 6×HA fragment

PCR2-TGME49_245485-Rv	CCGGATTCGTCCACAGCCAC	Detect the replacement of C-terminal TGME49_245485 by 6×HA fragment
sgRNA-TGME49_224620-Tag-Fw	GGTAGGACGCCTCTATAATAGTTTTAGAGCTAGAAATAGC	Construct the CRISPR plasmid for tagging TGME49_224620 with 6×HA
TGME49_224620-HR-Fw	GAAGGCCACGACAAATTCGTACTCCAACATCTGAACCCTTGGGCTAGCAAGGGCTCGGG	Amplify 6HA-DHFR fragment with the homologous fragment of *TGME49_224620*
TGME49_224620-HR-Rv	TGAGGGCCGAATCTGGCGGGTAGTATGTTTTGAACGCCGTATATACGACTCACTATAGG	Amplify 6HA-DHFR fragment with the homologous fragment of *TGME49_224620*
PCR1/2-TGME49_224620-Fw	GGCTGGCATTCCTGTCGTCATT	Detect the replacement of C-terminal TGME49_224620 by 6×HA fragment
PCR2-TGME49_224620-Rv	CCACCGAGCTGCGCTCCCTATA	Detect the replacement of C-terminal TGME49_224620 by 6×HA fragment
