# Supplementary material for: Functional Characterization of 11 Tentative Microneme Proteins in Type I RH Strain of Toxoplasma gondii Using the CRISPR-Cas9 System
Source: Animals (Basel). 2024 Sep 1;14(17):2543. doi: 10.3390/ani14172543 (PMC11394663; doi:10.3390/ani14172543)
Supplement: Supplementary file 1 [file animals-14-02543-s001.zip › Supplementary Table 2.pdf]

**Supplementary Table S2** Primers used in the construction of *T. gondii* RHΔ*GOI* strains.

| **Primer** | **Sequence (5′-3′)** | **Use** |
| --- | --- | --- |
| sgRNA-TGME49_243930-KO-Fw | GCAGACGGAGTTGTCCGGCGGTTTTAGAGCTAGAAATAGC | Construct the CRISPR plasmid for deleting *TGME49_243930* |
| U5-TGME49_243930-Gibson-Fw | GGTTTTCCCAGTCACGACGTTTGTGCCGATTCATTAGGTT | Amplify the 5' homologous arms of *TGME49_243930* to construct the pUPRT-DHFR-D plasmid |
| U5-TGME49_243930-Gibson-Rv | GGATTTACAGCCTGGCGAAGCTTGCTCACTGAGGACGGTGTC | Amplify the 5' homologous arms of *TGME49_243930* to construct the pUPRT-DHFR-D plasmid |
| U3-TGME49_243930-Gibson-Fw | CTATGCACTTGCAGGATGAATTCACCACTCGGGTGAGCATT | Amplify the 3' homologous arms of *TGME49_243930* to construct the pUPRT-DHFR-D plasmid |
| U3-TGME49_243930-Gibson-Rv | GAGCGGATAACAATTTCACAGCCATCATCGTTCGTCCT | Amplify the 3' homologous arms of *TGME49_243930* to construct the pUPRT-DHFR-D plasmid |
| DHFR-Gibson-Fw | AAGCTTCGCCAGGCTGTAAATCC | Amplify the DHFR fragment to construct the pUPRT-DHFR-D plasmid |
| DHFR-Gibson-Rv | GAATTCATCCTGCAAGTGCATAG | Amplify the DHFR fragment to construct the pUPRT-DHFR-D plasmid |
| pUC19-Gibson-Fw | TGTGAAATTGTTATCCGCTC | Amplify the pUC19 fragment to construct the pUPRT-DHFR-D plasmid |
| pUC19-Gibson-Rv | AACGTCGTGACTGGGAAAACC | Amplify the pUC19 fragment to construct the pUPRT-DHFR-D plasmid |
| PCR3-TGME49_243930-Fw | ATCGATGCTGCAGCGATCGAAGCT | Detect the insertion of 5' homologous fragment of *TGME49_243930* in PCR3 |
| PCR3-DHFR-Rv | GCCAAAGTAGAAAGGAATTAGCAT | Detect the insertion of 5' homologous fragment of *TGME49_243930* in PCR3 |
| PCR4-TGME49_243930-Fw | ACTGTGGATTTGCGTCTT | Detect the deletion of *TGME49_243930* in PCR4 |
| PCR4-TGME49_243930-Rv | GTTTCGGTGAGTTTCTGTG | Detect the deletion of *TGME49_243930* in PCR4 |
| PCR5-DHFR-Fw | TGACGCAGATGTGCGTGTATCCAC | Detect the insertion of 3' homologous fragment of *TGME49_243930* in PCR5 |
| PCR5-TGME49_243930-Rv | TGGATTCTGCAGCAAGGTGACTCT | Detect the insertion of 3' homologous fragment of *TGME49_243930* in PCR5 |
| sgRNA-TGME49_200270-KO-Fw | GCATTGTTCTTCTCCTGAGAGTTTTAGAGCTAGAAATAGC | Construct the CRISPR plasmid for deleting *TGME49_200270* |
| U5-TGME49_200270-Fw | GGTTTTCCCAGTCACGACGTTGAAACGGGTGATACGAAGG | Amplify the 5' homologous arms of *TGME49_200270* to construct the pUPRT-DHFR-D plasmid |
| U5-TGME49_200270-Rv | GGATTTACAGCCTGGCGAAGCTTCGGCACTATTATGGAGCAA | Amplify the 5' homologous arms of *TGME49_200270* to construct the pUPRT-DHFR-D plasmid |
| U3-TGME49_200270-Fw | CTATGCACTTGCAGGATGAATTCATAAGTGCCTCAACGACG | Amplify the 3' homologous arms of *TGME49_200270* to construct the pUPRT-DHFR-D plasmid |
| U3-TGME49_200270-Rv | GAGCGGATAACAATTTCACAGTGGCGATTGTAGAAAGC | Amplify the 3' homologous arms of *TGME49_2002700* to construct the pUPRT-DHFR-D plasmid |
| PCR3-TGME49_200270-Fw | AAAGACACAGCTCTTCCTCCGCGT | Detect the insertion of 5' homologous fragment of *TGME49_200270* in PCR3 |
| PCR4-TGME49_200270-Fw | TAGTCAACGCCCACTCTTT | Detect the deletion of *TGME49_200270* in PCR4 |
| PCR4-TGME49_200270-Rv | GGACCTGCGACATACTCATT | Detect the deletion of *TGME49_200270* in PCR4 |
| PCR5-TGME49_200270-Rv | TATCGTCCCTTCGTGGTCACTGCT | Detect the insertion of 3' homologous fragment of *TGME49_200270* in PCR5 |

| | | |
|---|---|---|
| sgRNA-TGME49_273320-KO-Fw | GGAGGAAGCCGACATCGAGGGTTTTAGAGCTAGAAATAGC | Construct the CRISPR plasmid for deleting *TGME49_273320* |
| U5-TGME49_273320-Fw | GGTTTTCCCAGTCACGACGTTGCGGCGACCACAAAGAGT | Amplify the 5' homologous arms of *TGME49_273320* to construct the pUPRT-DHFR-D plasmid |
| U5-TGME49_273320-Rv | GGATTTACAGCCTGGCGAAGCTTGACCCACAAGACAGATGAAACA | Amplify the 5' homologous arms of *TGME49_273320* to construct the pUPRT-DHFR-D plasmid |
| U3-TGME49_273320-Fw | CTATGCACTTGCAGGATGAATTCAGAACAATCAGGGTGGGT | Amplify the 3' homologous arms of *TGME49_273320* to construct the pUPRT-DHFR-D plasmid |
| U3-TGME49_273320-Rv | GAGCGGATAACAATTTCACAGCTGTCTTGATAGGTGGC | Amplify the 3' homologous arms of *TGME49_273320* to construct the pUPRT-DHFR-D plasmid |
| PCR3-TGME49_273320-Fw | GTTCTCTGTACACACCAGGCTCAA | Detect the insertion of 5' homologous fragment of *TGME49_273320* in PCR3 |
| PCR4-TGME49_273320-Fw | GACTTGCCTGTCTCCACC | Detect the deletion of *TGME49_273320* in PCR4 |
| PCR4-TGME49_273320-Rv | GAAACCGATACATCCATTCT | Detect the deletion of *TGME49_273320* in PCR4 |
| PCR5-TGME49_273320-Rv | ATCACAACTCTGGCTTCAAAGGGG | Detect the insertion of 3' homologous fragment of *TGME49_273320* in PCR5 |
| sgRNA-TGME49_243790-KO-Fw | GTCCGCTGGCACCGAACTGAGTTTTAGAGCTAGAAATAGC | Construct the CRISPR plasmid for deleting *TGME49_243790* |
| U5-TGME49_243790-Fw | GGTTTTCCCAGTCACGACGTTGGTGTAATTCACCCAGCAT | Amplify the 5' homologous arms of *TGME49_243790* to construct the pUPRT-DHFR-D plasmid |
| U5-TGME49_243790-Rv | GGATTTACAGCCTGGCGAAGCTTGACAAAGACAGCGAGCAGA | Amplify the 5' homologous arms of *TGME49_243790* to construct the pUPRT-DHFR-D plasmid |
| U3-TGME49_243790-Fw | CTATGCACTTGCAGGATGAATTCCCACCGTACCCGTCGTTCA | Amplify the 3' homologous arms of *TGME49_243790* to construct the pUPRT-DHFR-D plasmid |
| U3-TGME49_243790-Rv | GAGCGGATAACAATTTCACATTTCGCCGTCTCGCCTCT | Amplify the 3' homologous arms of *TGME49_243790* to construct the pUPRT-DHFR-D plasmid |
| PCR3-TGME49_243790-Fw | TCAGAAGGCGGAAAAGGAAGGATC | Detect the insertion of 5' homologous fragment of *TGME49_243790* in PCR3 |
| PCR4-TGME49_243790-Fw | CACTTCTCGCAGTTGTCGC | Detect the deletion of *TGME49_243790* in PCR4 |
| PCR4-TGME49_243790-Rv | GGTGGCTTCGCATTCTCC | Detect the deletion of *TGME49_243790* in PCR4 |
| PCR5-TGME49_243790-Rv | TTCCTGATTATCACCATAGCGGCG | Detect the insertion of 3' homologous fragment of *TGME49_243790* in PCR5 |
| sgRNA-TGME49_287040-KO-Fw | GAGACCGGTGAGAGCAACAGGTTTTAGAGCTAGAAATAGC | Construct the CRISPR plasmid for deleting *TGME49_287040* |
| U5-TGME49_287040-Fw | GGTTTTCCCAGTCACGACGTTCGGCTGACAGTCGATGTT | Amplify the 5' homologous arms of *TGME49_287040* to construct the pUPRT-DHFR-D plasmid |
| U5-TGME49_287040-Rv | GGATTTACAGCCTGGCGAAGCTTGTGCTTACCACGCGGAAT | Amplify the 5' homologous arms of *TGME49_287040* to construct the pUPRT-DHFR-D plasmid |
| U3-TGME49_287040-Fw | CTATGCACTTGCAGGATGAATTCGCGTGCCTTCGTGACTGCT | Amplify the 3' homologous arms of *TGME49_287040* to construct the pUPRT-DHFR-D plasmid |
| U3-TGME49_287040-Rv | GAGCGGATAACAATTTCACATTACCGCCATCGACCTCCC | Amplify the 3' homologous arms of *TGME49_287040* to construct the pUPRT-DHFR-D plasmid |
| PCR3-TGME49_287040-Fw | TTCCGGCGGATGTTTAACGTCTCC | Detect the insertion of 5' homologous fragment of *TGME49_287040* in PCR3 |
| PCR4-TGME49_287040-Fw | GGAAGGAAGCGGAGAAGC | Detect the deletion of *TGME49_287040* in PCR4 |
| PCR4-TGME49_287040-Rv | TGCCCGTCGTGGTAGATG | Detect the deletion of *TGME49_287040* in PCR4 |
| PCR5-TGME49_287040-Fw | GTCAGTCTGTTTCCGCAGCAGAAA | Detect the insertion of 3' homologous fragment of *TGME49_287040* in PCR5 |

| | | |
|---|---|---|
| sgRNA-TGME49_261710-KO-Fw | GCCGCCGCCGGAGCCTGACGGTTTTAGAGCTAGAAATAGC | Construct the CRISPR plasmid for deleting *TGME49_261710* |
| U5-TGME49_261710-Fw | GGTTTTCCCAGTCACGACGTTCCGCTTGTTGCTGGTCTGTGC | Amplify the 5' homologous arms of *TGME49_261710* to construct the pUPRT-DHFR-D plasmid |
| U5-TGME49_261710-Rv | GGATTTACAGCCTGGCGAAGCTTTTCCACGAAGAAACCCACTTGACA | Amplify the 5' homologous arms of *TGME49_261710* to construct the pUPRT-DHFR-D plasmid |
| U3-TGME49_261710-Fw | CTATGCACTTGCAGGATGAATTCGCAGCGTGTTCATTCCATCTTTCG | Amplify the 3' homologous arms of *TGME49_261710* to construct the pUPRT-DHFR-D plasmid |
| U3-TGME49_261710-Rv | GAGCGGATAACAATTTCACACGCCCCACTTAGGACAAGCAAA | Amplify the 3' homologous arms of *TGME49_261710* to construct the pUPRT-DHFR-D plasmid |
| PCR3-TGME49_261710-Fw | ACAGACCATTGGTGACAGACCAAC | Detect the insertion of 5' homologous fragment of *TGME49_261710* in PCR3 |
| PCR4-TGME49_261710-Fw | TGCCGCTTTGCAGCCACCTT | Detect the deletion of *TGME49_261710* in PCR4 |
| PCR4-TGME49_261710-Rv | TGCCACCAACTCCTGTTATTCCGT | Detect the deletion of *TGME49_261710* in PCR4 |
| PCR5-TGME49_261710-Rv | ACGAGAGATTCGTTGGAAGCGACG | Detect the insertion of 3' homologous fragment of *TGME49_261710* in PCR5 |
| sgRNA-TGME49_272380-KO-Fw | GAATTCACCACGTGTCGCCGGTTTTAGAGCTAGAAATAGC | Construct the CRISPR plasmid for deleting *TGME49_272380* |
| U5-TGME49_272380-Fw | GGTTTTCCCAGTCACGACGTTTTCACCCGCTGCTTCAATCCC | Amplify the 5' homologous arms of *TGME49_272380* to construct the pUPRT-DHFR-D plasmid |
| U5-TGME49_272380-Rv | GGATTTACAGCCTGGCGAAGCTTTCTACCCCAACCCTGCTTCGACTA | Amplify the 5' homologous arms of *TGME49_272380* to construct the pUPRT-DHFR-D plasmid |
| U3-TGME49_272380-Fw | CTATGCACTTGCAGGATGAATTCTCAAGGAACGAAATGCGGGTGA | Amplify the 3' homologous arms of *TGME49_272380* to construct the pUPRT-DHFR-D plasmid |
| U3-TGME49_272380-Rv | GAGCGGATAACAATTTCACAGCGGGATTAGGCGTGGAAGC | Amplify the 3' homologous arms of *TGME49_272380* to construct the pUPRT-DHFR-D plasmid |
| PCR3-TGME49_272380-Fw | GAGGCTGGCGAGGTCACAAACATA | Detect the insertion of 5' homologous fragment of *TGME49_272380* in PCR3 |
| PCR4-TGME49_272380-Fw | GGTGGTTCCTGGTCGGATCTTTG | Detect the deletion of *TGME49_272380* in PCR4 |
| PCR4-TGME49_272380-Rv | TGCGTTTCCCCGTTAGTTGGTG | Detect the deletion of *TGME49_272380* in PCR4 |
| PCR5-TGME49_272380-Rv | TGTAACCCTTTGGCCTATCCACAC | Detect the insertion of 3' homologous fragment of *TGME49_272380* in PCR5 |
| sgRNA-TGME49_205680-KO-Fw | GTATCAGCTGGACAGAACCAGTTTTAGAGCTAGAAATAGC | Construct the CRISPR plasmid for deleting *TGME49_205680* |
| U5-TGME49_205680-Fw | GGTTTTCCCAGTCACGACGTTAGTGGCATGAAGTGATGCGGATTT | Amplify the 5' homologous arms of *TGME49_205680* to construct the pUPRT-DHFR-D plasmid |
| U5-TGME49_205680-Rv | GGATTTACAGCCTGGCGAAGCTTGTCAGCGTCTGCGTCGGTGTAG | Amplify the 5' homologous arms of *TGME49_205680* to construct the pUPRT-DHFR-D plasmid |
| U3-TGME49_205680-Fw | CTATGCACTTGCAGGATGAATTCTACAAGCAGACGATTGCCTGGGTC | Amplify the 3' homologous arms of *TGME49_205680* to construct the pUPRT-DHFR-D plasmid |
| U3-TGME49_205680-Rv | GAGCGGATAACAATTTCACAGCGAGCACGCCAAAGGAAGG | Amplify the 3' homologous arms of *TGME49_2056800* to construct the pUPRT-DHFR-D plasmid |
| PCR3-TGME49_205680-Fw | TTAGCATGCCAGCTTCTCTCTGTG | Detect the insertion of 5' homologous fragment of *TGME49_205680* in PCR3 |
| PCR4-TGME49_205680-Fw | ACCTATTATGAATCCTGCGTCCCG | Detect the deletion of *TGME49_205680* in PCR4 |
| PCR4-TGME49_205680-Rv | CAGCGTTGACTGCTGGCTGTTCT | Detect the deletion of *TGME49_205680* in PCR4 |
| PCR5-TGME49_205680-Rv | GAGGAGCATATCCAGGAGAAGTTC | Detect the insertion of 3' homologous fragment of *TGME49_205680* in PCR5 |

| | | |
|---|---|---|
| sgRNA-TGME49_304490-KO-Fw | GATTCTCTCGCTGGTAGCAAGTTTTAGAGCTAGAAATAGC | Construct the CRISPR plasmid for deleting *TGME49_304490* |
| U5-TGME49_304490-Fw | GGTTTTCCCAGTCACGACGTTTTTTCCGACCCCGTGCTTGA | Amplify the 5' homologous arms of *TGME49_304490* to construct the pUPRT-DHFR-D plasmid |
| U5-TGME49_304490-Rv | GGATTTACAGCCTGGCGAAGCTTGCCCTACGAAGATCGCCGAATC | Amplify the 5' homologous arms of *TGME49_304490* to construct the pUPRT-DHFR-D plasmid |
| U3-TGME49_304490-Fw | CTATGCACTTGCAGGATGAATTCCTGCTTCTGTCAGGCGATGGG | Amplify the 3' homologous arms of *TGME49_304490* to construct the pUPRT-DHFR-D plasmid |
| U3-TGME49_304490-Rv | GAGCGGATAACAATTTCACATGTCCGAAACACGAAATCAACTGC | Amplify the 3' homologous arms of *TGME49_304490* to construct the pUPRT-DHFR-D plasmid |
| PCR3-TGME49_304490-Fw | CTGACCGGATAAGCTAGCCCAGTT | Detect the insertion of 5' homologous fragment of *TGME49_304490* in PCR3 |
| PCR4-TGME49_304490-Fw | TTTCCTGACCCGAACATCCAACA | Detect the deletion of *TGME49_304490* in PCR4 |
| PCR4-TGME49_304490-Rv | GGCTATGCTACCAACGCCAAGACT | Detect the deletion of *TGME49_304490* in PCR4 |
| PCR5-TGME49_304490-Rv | TACTTTTTACGCACTGCGGGTGTG | Detect the insertion of 3' homologous fragment of *TGME49_304490* in PCR5 |
| sgRNA-TGME49_245485-KO-Fw | GGTAGAAGCTTGCCTCGTGAGTTTTAGAGCTAGAAATAGC | Construct the CRISPR plasmid for deleting *TGME49_245485* |
| U5-TGME49_245485-Fw | GGTTTTCCCAGTCACGACGTTACCGCTACGATTGTCCTCACGTC | Amplify the 5' homologous arms of *TGME49_245485* to construct the pUPRT-DHFR-D plasmid |
| U5-TGME49_245485-Rv | GGATTTACAGCCTGGCGAAGCTTACCCTTCGATTCTGCCCTCATCT | Amplify the 5' homologous arms of *TGME49_245485* to construct the pUPRT-DHFR-D plasmid |
| U3-TGME49_245485-Fw | CTATGCACTTGCAGGATGAATTCTGCCTTTGCCTGTTCGCTGTG | Amplify the 3' homologous arms of *TGME49_245485* to construct the pUPRT-DHFR-D plasmid |
| U3-TGME49_245485-Rv | GAGCGGATAACAATTTCACAGCTGAAACCTGCACTAGCCATCCT | Amplify the 3' homologous arms of *TGME49_245485* to construct the pUPRT-DHFR-D plasmid |
| PCR3-TGME49_245485-Fw | ACATCTGTGCGGGCAACAAGCAGT | Detect the insertion of 5' homologous fragment of *TGME49_245485* in PCR3 |
| PCR4-TGME49_245485-Fw | GACACCAGCCCCTGTGGAACG | Detect the deletion of *TGME49_245485* in PCR4 |
| PCR4-TGME49_245485-Rv | CGCTGAGTCGGAACCTGTTTTCTT | Detect the deletion of *TGME49_245485* in PCR4 |
| PCR5-TGME49_245485-Rv | AGTTTCGCCGTCACTCTCTCCTGT | Detect the insertion of 3' homologous fragment of *TGME49_245485* in PCR5 |
| sgRNA-TGME49_224620-KO-Fw | GCGTCAAGTTGACGACACCAGTTTTAGAGCTAGAAATAGC | Construct the CRISPR plasmid for deleting *TGME49_224620* |
| U5-TGME49_224620-Fw | GGTTTTCCCAGTCACGACGTTTGAATCTCATCGGAACGCTCTGTC | Amplify the 5' homologous arms of *TGME49_224620* to construct the pUPRT-DHFR-D plasmid |
| U5-TGME49_224620-Rv | GGATTTACAGCCTGGCGAAGCTTGCAACTCCAATCACCCGCAAAT | Amplify the 5' homologous arms of *TGME49_224620* to construct the pUPRT-DHFR-D plasmid |
| U3-TGME49_224620-Fw | CTATGCACTTGCAGGATGAATTCTCGGACCTTTGCGTGGTGTTG | Amplify the 3' homologous arms of *TGME49_224620* to construct the pUPRT-DHFR-D plasmid |
| U3-TGME49_224620-Rv | GAGCGGATAACAATTTCACAGACTGGAGGAAGGGGAAGGAAGC | Amplify the 3' homologous arms of *TGME49_224620* to construct the pUPRT-DHFR-D plasmid |
| PCR3-TGME49_224620-Fw | CGAGTGCGTCGAAGAAACATGAGG | Detect the insertion of 5' homologous fragment of *TGME49_224620* in PCR3 |
| PCR4-TGME49_224620-Fw | TTTCTGTCGGGTTTTCTCCGATGT | Detect the deletion of *TGME49_224620* in PCR4 |
| PCR4-TGME49_224620-Rv | GGGCTGGTCACTCAGGCTTCC | Detect the deletion of *TGME49_224620* in PCR4 |
| PCR5-TGME49_224620-Rv | GCCGTGGGACAATAGCTGTAATTG | Detect the insertion of 3' homologous fragment of *TGME49_224620* in PCR5 |
