# Supplementary figures and images for: Functional Characterization of 11 Tentative Microneme Proteins in Type I RH Strain of Toxoplasma gondii Using the CRISPR-Cas9 System
Source: Animals (Basel). 2024 Sep 1;14(17):2543. doi: 10.3390/ani14172543 (PMC11394663; doi:10.3390/ani14172543)

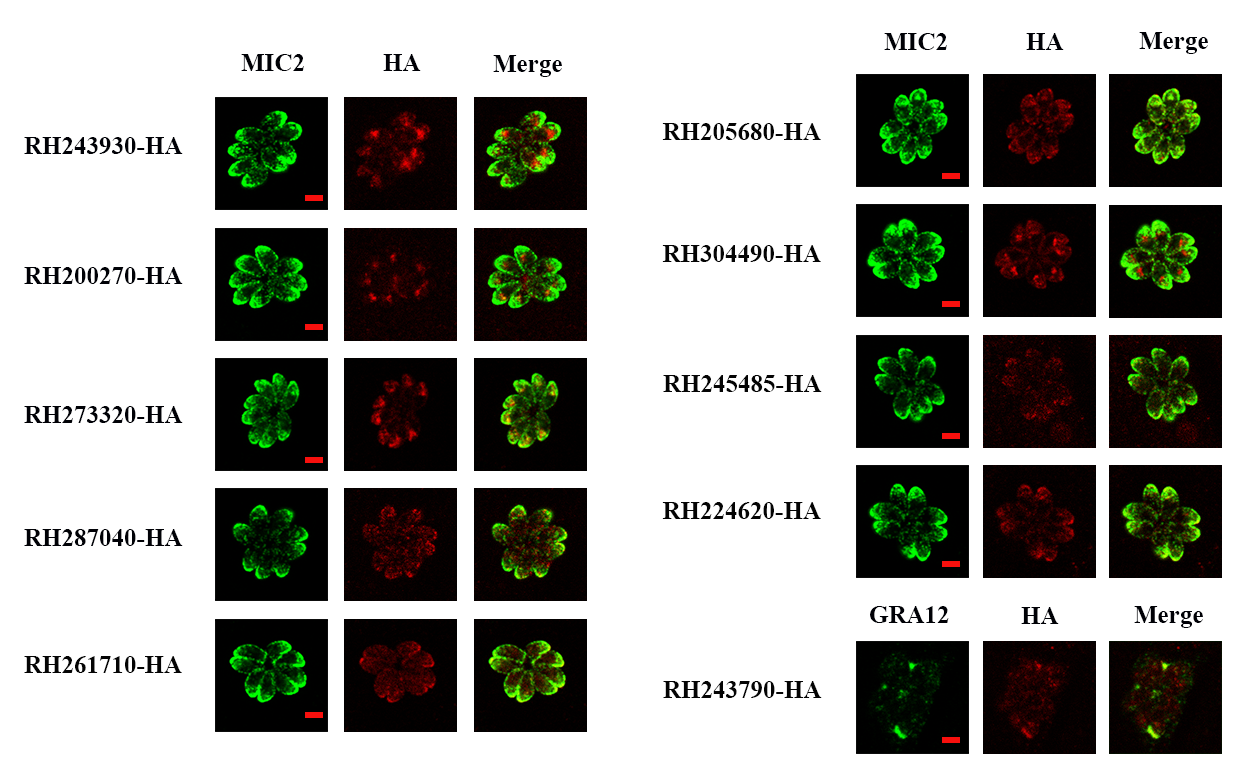

Supplement: Supplementary file 1 [file animals-14-02543-s001.zip › Fig.S1.tif]
